# Characterization of selected LDLR substitutions in patients with familial hypercholesterolemia

**DOI:** 10.1016/j.athplu.2025.11.001

**Published:** 2025-11-18

**Authors:** Monika Targońska, Anna Janaszak-Jasiecka, Magdalena Chmara, Monika Żuk, Leszek Kalinowski, Krzysztof Waleron, Jacek Jasiecki, Bartosz Wasąg

**Affiliations:** a Department of Biology and Medical Genetics, Medical University of Gdańsk, 80-210, Gdańsk, Poland; b Department of Medical Laboratory Diagnostics—Fahrenheit Biobank BBMRI.pl, Medical University of Gdańsk, 80-211, Gdańsk, Poland; c Laboratory of Clinical Genetics, University Clinical Centre, 80-952, Gdańsk, Poland; d Center of Translational Medicine, Medical University of Gdańsk, 80-210, Gdańsk, Poland; e BioTechMed Centre/Department of Mechanics of Materials and Structures, Gdansk University of Technology, 80-233, Gdańsk, Poland; f Department of Pharmaceutical Microbiology, Faculty of Pharmacy, Medical University of Gdańsk, 80-416, Gdańsk, Poland

**Keywords:** Familial Hypercholesterolemia (FH), Low-Density Lipoprotein (LDL), Low-Density Lipoprotein Cholesterol (LDL-C), LDL Receptor (LDLR), Variants classification, HEK293T-*ldlr*G1

## Abstract

**Background and aims:**

Familial hypercholesterolemia is a genetic disorder caused by pathogenic or likely pathogenic variants in four key genes: *LDLR*, *APOB*, *PCSK9,* and *APOE*. It leads to elevated levels of low-density lipoprotein cholesterol in the bloodstream and significantly increases the risk of coronary artery disease. This study aimed to functionally characterize *LDLR* variants identified in Polish FH patients. Experimental data were used to learn about variants' phenotypes and incorporate them into the ACMG/AMP variant classification framework.

**Methods:**

The functional analysis was performed using the HEK293T-*ldlr*G1 cells with the expression vectors pTetRedLDLR carrying the mutated *LDLR* gene variants. Receptor expression was evaluated using Western blot and immunofluorescence. The low-density lipoprotein uptake and ligand binding capacity were examined with fluorescent dye-labeled LDL by confocal microscopy. A functional study was performed to analyze the variants under assessment and compare them to known benign and pathogenic control variants.

**Results:**

The experimental study revealed an impaired activity of the c.662A > G p. (Asp221Gly), c.1775G > A p. (Gly592Glu), and c.2483delA p. (Tyr828Phefs∗101) *LDLR* variants, classifying them as functionally abnormal. In contrast, *in vitro* activity assessment of the c.91G > A p. (Glu31Lys) *LDLR* variant showed fully functional low-density lipoprotein binding and uptake activities. These results suggested that c.91G > A p. (Glu31Lys) is unlikely to be a disease-causing variant.

**Conclusions:**

The results provide functional evidence for the activity of selected *LDLR* variants in a cellular model based on confocal techniques that meets the ACMG/AMP variant classification criteria. These findings highlight the importance of *in vitro* assays in evaluating the functional impact of *LDLR* variants and contribute valuable insights for clinical interpretation and genetic counseling.

## Highlights

• Functional analysis using single-cell assays revealed that three *LDLR* variants (p.Asp221Gly, p.Gly592Glu, p.Tyr828Phefs∗101) show impaired receptor activity due to reduced expression, ligand binding, internalization defects, or a stop-loss function, supporting their pathogenic role in familial hypercholesterolemia.
• The p.Glu31Lys variant demonstrated normal LDL-C binding and uptake, suggesting it is likely benign and not disease-causing.
• This study emphasizes the importance of *in vitro* functional assays for classifying variants of uncertain significance (VUS) within the ACMG/AMP framework, aiding genetic diagnosis and counseling.

## 1 Introduction

Familial hypercholesterolemia (FH), with an autosomal semi-dominant inheritance pattern, is due to a monogenic defect in LDL receptor (*LDLR*), apolipoprotein B (*APOB*), proprotein convertase subtilisin/kexin type 9 (*PCSK9*), or apolipoprotein E (*APOE*) genes, which encode proteins involved in cholesterol metabolism [1,2]. It contributes to an increased level of low-density lipoprotein cholesterol (LDL-C) in the blood and increased incidence of coronary artery disease (CAD). Alterations of the *LDLR* gene constitute over 90 % of FH cases and include over 4500 variants (ClinVar Database) [[3], [4], [5]]. *LDLR* variants may impact gene expression and maturation, cell membrane localization, ligand binding, internalization, or receptor recycling. Consequently, insufficient low-density lipoprotein (LDL) uptake from the bloodstream, leads to elevated circulating LDL levels in the plasma, and their subsequent deposition in the arterial walls ultimately drives the development of atherosclerosis [[6], [7], [8], [9], [10]].

In 2022, the Clinical Genome Resource Familial Hypercholesterolemia (FH) Variant Curation Expert Panel optimized the classification of variants detected in FH individuals. It underlined the importance of experimental studies in this process. Functional characterization of LDLR activity should be performed in heterologous cells (with no endogenous LDLR) transfected with mutant plasmids. Comprehensive analysis should include the entire LDLR cycle: LDLR expression, LDL binding, and LDL internalization. The LDLR activity threshold >90 % compared to the wild-type meets the benign functional study criteria. On the other hand, the LDLR activity threshold <70 % compared to the wild-type constitutes strong evidence for the pathogenicity of the variant under assessment (VUA) [[11], [12], [13], [14]]. However, only 10 % of detected *LDLR* variants have undergone functional analysis [15].

We previously developed the LDLR-defective HEK293T-*ldlr*G1 cell line, established by a CRISPR/Cas9-mediated luciferase–puromycin knock-in with silenced endogenous *LDLR* gene expression to determine the effect of genetic variants of the *LDLR* gene on receptor activity. The cells were transfected by expression pTetRedLDLR vectors carrying *LDLR* variants and the *DsRed2* reporter gene, which induced the LDLR variants overexpression and red fluorescence of the dsRED2 protein [16].

This study represents the functional analysis of selected *LDLR* gene variants detected in patients with the hypercholesterolemia phenotype. A study of the Polish population identified several variants in the *LDLR* gene, out of which c.662A > G p. (Asp221Gly), c.1775G > A p. (Gly592Glu), and exons 4–8 duplication accounted for approximately 36 % of all the variants [17]. The above missense variants were selected for being the most prevalent among the group of variants still lacking functional characterization at criterium PS3 at level 1 assay, according to ClinGen Familial Hypercholesterolemia Expert Panel Specifications to the ACMG/AMP Variant Classification Guidelines Version 1.2. Moreover, the missense variant of unknown significance, c.91G > A p. (Glu31Lys), was detected in three unrelated patients with possible (5 points), probable (6 points), and definite (8 points) FH diagnosis according to The Dutch Lipid Clinical Network criteria. This variant was studied here. Sequencing of the *LDLR* gene revealed a stop-loss mutation c.2483delA p. (Tyr828Phefs∗101) in one family. Variants leading to premature protein termination and loss of essential functional domains are typically classified as pathogenic. However, the impact of frameshift variants resulting in an extended incorrect terminus is unclear. We used validated functional assays to perform a systematic functional assessment of the c.2483delA p. (Tyr828Phefs∗101) variant, leading to transcript extension beyond the regular stop codon. The above variants were compared to wild-type LDLR as well as to pathogenic control variants c.661G > T p. (Asp221Tyr) [18], c.1216C > T p. (Arg406Trp) [19], and benign control variant c.2177C > T p. (Thr726Ile) [20] by the use of HEK293T-*ldlr*G1 cell line.

## 2 Materials and methods

### 2.1 Cloning of expression vectors carrying *LDLR* gene variants

The bicistronic expression vectors carrying *LDLR* variants and the *DsRed* gene were cloned into between the *EcoRI* and *PstI* sites of the pTetOne expression vector by the In-Fusion® HD Cloning system (Takara Bio Inc., Shiga, Japan), as described in detail by Jasicki et al. [16,21]. Vector pCMV6-LDLR (Origene, Rockville, MD, USA) with a full-length cDNA of *LDLR* (NM_000527.2) was template for *LDLR* variants, exept for c.2483delA p. (Tyr828Phefs∗101) variant. In ordert to clone c.2483delA p. (Tyr828Phefs∗101) variant, we isolated mRNA from HUVEC cell line (Catalog #: C2519A, Lonza; Basel, Switzerland) and reverse transcribed mRNA into cDNA, which included 3′UTR of *LDLR.* The obtained cDNA constituted template for cloning c.2483delA p. (Tyr828Phefs∗101) variant. The PCR primer pairs and templates used to create variants are listed in Supplementary Materials (S1). Obtained vectors were transformed into *E. coli Stellar™* and isolated from the bacteria. The vectors were confirmed by Sanger sequencing.

### 2.2 Cell culture and transfection

HEK293T-*ldlr*G1 cells were cultured in 40 % MEM, 40 % F12, 10 % William's E medium supplemented with 10 % fetal bovine serum, 2 mmol/L L-glutamine, 100 units/mL penicillin, 100 μg/mL streptomycin (Thermo Fisher Scientific, USA), and 5 μg/ml of the puromycin. The cells were transfected with the expression vectors by the JetPRIME Transfection Reagent (plasmid/jetPRIME ratio 1:2) (Polyplus, Illkirch, France). The medium was replaced by a fresh one with doxycycline (DOX) (10, 100 ng/mL) after 4 h. The transfection efficiency was assessed 48 h post-transfection as red fluorescence of DsRed2 under a fluorescence microscope (Supplementary Materials, S2), and cells underwent molecular tests.

### 2.3 Western blot analysis

Transfected cells cultured in medium supplemented with 100 ng/mL DOX were harvested 48 h after transfection. Cell lysates were loaded onto Mini-PROTEAN 4–15 % precast TGX Stain-Free gels (Bio-Rad, Hercules, CA, USA). The electrophoresis was conducted for 90 min at 100 V. Proteins were blotted onto a PVDF membrane using a wet transfer system for 1 h at 100 V. The membrane was blocked with TBST buffer (20 mM Tris 7,5 pH, 150 mM NaCl, 0,1 % Tween 20) with 3 % skim milk for 1 h at 4 °C. The proteins were detected following using the primary mouse monoclonal antibodies: anti-LDLR antibody (1:1000) (Cat. No. 61087, Progen Biotechnik GmbH; Germany), mouse monoclonal anti-β-actin antibody (1:10000) (Cat. No. ab6276, Abcam, Cambridge, UK), and rabbit polyclonal DsRed2 antibody (1:1000) (Cat. No. 632496, Takara Bio Inc., Shiga, Japan). Corresponding HRP-conjugated secondary antibodies (anti-mouse and anti-rabbit IgG 1:3000 Bio-Rad, Hercules, CA, USA) were used for chemiluminescence detection with the Clarity Western ECL Substrate chemiluminescence kit (Bio-Rad, Hercules, CA, USA) in the ChemiDoc Touch Imaging system (Bio-Rad, Hercules, CA, USA). To enable sequential detection, the membrane was stripped using Restore™ Western Blot Stripping Buffer (Thermo Scientific, Rockford, USA): first to remove the LDLR signal for subsequent detection of DsRed2, and then again to detect β-actin. Densitometric analysis of LDLR variant expression was performed using Image Lab software (Version 6.1; Bio-Rad; USA). DsRed2 levels indicated transfection efficiency and served as a reporter protein for normalization (Supplementary Materials, S2).

### 2.4 Immunofluorescence analysis

Transfected cells were cultured in the medium supplemented with 10 ng/mL DOX in Cell Carrier™-96 Ultra plates (PerkinElmer; USA) for 48 h. After incubation, cells were fixed with 4 % paraformaldehyde and blocked with 1 % BSA in DPBS buffer (Sigma; USA). The surface expression of LDLR was detected using a mouse monoclonal anti-LDLR primary antibody (1:100) (Cat. No. 61087, Progen Biotechnik GmbH, Heidelberg; Germany) followed by an Alexa Fluor™ 488- conjugated goat anti-mouse secondary antibody (1:2000) (Cat. No. A11001, Thermo Fisher Scientific; Waltham; MA; USA). Nuclei were counterstained with PureBlu™ Hoechst 33342 (Bio-Rad; USA). Confocal microscopy revealed LDLR as green fluorescence, DsRed2 as yellow fluorescence, and the nuclei as blue. Only yellow cells were selected for quantification of the LDLR variant expression on the cell surface, and analysis was performed using ImageJ software.

### 2.5 LDL uptake assay

Cells transfected with expression vectors were cultured in a medium supplemented with 10 ng/mL DOX in the Cell Carrier™-96 Ultra plates for 48 h. Then, the cells were incubated in a starvation medium (50 % MEM, 50 % William's E, 0,3 % BSA, 100 units/mL penicillin, and 100 μg/mL streptomycin) for 2 h at 37 °C under 5 % CO_2_. The pH-sensitive fluorescent dye pHrodo™ Green-LDL (Thermo Fisher Scientific, Carlsbad, USA) was added to the medium at a final concentration of 5 μg/mL. The cells were immediately transferred to the Opera Phenix High Content Screening System, and images were acquired every 30 min for 150 min. The LDL uptake capacity by LDLR variants was quantified using ImageJ software. The method was based on the use of only transfected cells, visible as yellow cell in a confocal microscope. The uptake rate constant was determined as the slope of a linear function passing through the origin.

### 2.6 LDL binding assay

Transfected cells were cultured in a medium supplemented with 100 ng/mL DOX in Cell Carrier™-96 Ultra plates for 48 h. The cells were then incubated in a starvation medium for 2 h at 37 °C under 5 % CO_2_. Receptors were subsequently fixed with 0,5 % paraformaldehyde. Next, the fluorescent dye BODIPY™ FL LDL (Thermo Fisher Scientific, Carlsbad, USA) was added to the medium at a final concentration of 5 μg/mL, and the cells were incubated for 2 h at 4 °C. Nuclei were stained with PureBlu™ Hoechst 33342 (Bio-Rad; USA), washed with fresh starvation medium, and analyzed by confocal microscopy. The LDL binding capacity of the variants was quantified based on the intensity of green fluorescence of transfected cells using ImageJ software.

### 2.7 Confocal laser scanning microscopy

Images were acquired using the Opera Phenix High Content Screening System and Harmony 4.8 software (PerkinElmer, USA) with a 63× water immersion objective (NA 1.15). Alexa Fluor 488, Bodipy FL-labeled LDL, and pHrodo™ Green conjugate signals were visualized using a 488 nm bandpass excitation filter and 500–550 nm bandpass emission filter. The DsRed2 signal was detected with a 561 nm bandpass excitation filter and a 570–630 nm bandpass emission filter. PureBlu™ Hoechst 33342 signal was visualized with a 405 nm bandpass excitation filter and a 435–480 nm bandpass emission filter. All images were captured in 16-bit format at a resolution of 1080 x 1080 pixels. Exposure time and laser power were kept constant across all imaging sessions. Image acquisition and processing were performed using Harmony 4.8 software (PerkinElmer).

### 2.8 Patient's screening

The study has been approved by the Ethical Committee. Written informed consent was obtained from all participants. Fasting blood samples were collected from individuals. Analysis of the *LDLR* gene was performed using Sanger or next-generation sequencing.

### 2.9 Data analysis

The predicted effects of *LDLR* missense variants were assessed by open-access *in silico* tools: Rare Exome Variant Ensemble Learner (REVEL) [22] and Maximum Entropy Scan (MES) [23].

Confocal microscopy images were analyzed using ImageJ software (NIH, Bethesda, MD, USA). Images were converted to 8-bit images with an adjusted lower threshold of 30 and an upper threshold of 200 [24].

Quantitative data represent the mean of measurements with standard deviation (SD). Statistical significance of variants were compared to LDLR-WT using a two-tailed *t*-test in Microsoft Excel, with *p* < 0.05 [∗], *p* < 0,01 [∗∗], *p* < 0,001 [∗∗∗], p < 0,0001 [∗∗∗∗] considered statistically significant.

## 3 Results

Selected *LDLR* alterations detected in the Polish FH cohort were subjected to experimental studies using *LDLR*-defective HEK293T-*ldlr*G1 cell line transfected with expression pTetRedLDLR vector carrying *LDLR* variants. The data was compared to activity wild-type LDLR.

**Fig. 1:**
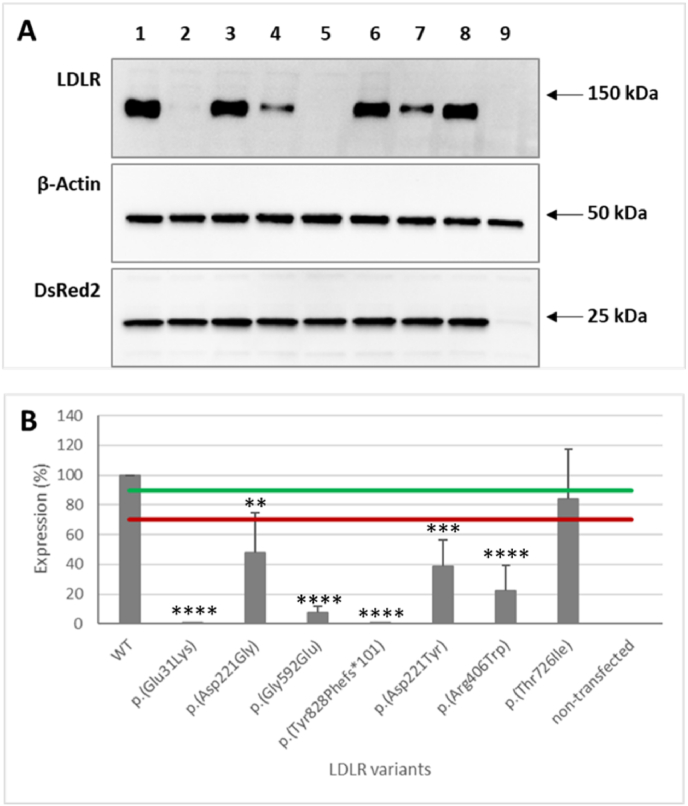
Cellular expression of the LDLR variants. HEK293T-*ldlr*G1 cells transfected with pTetRedLDLR expression vectors were cultured in the medium supplemented with 100 ng/mL doxycycline for 48 h. The cell lysates were loaded onto a gel at equal total protein concentrations and analyzed by Western blot. **A:** The 150 kDa bands represent mature form of LDLR wild-type (WT) (line 1), c.91G > A p. (Glu31Lys) (line 2), c.662 A > G p. (Asp221Gly) (line 3), c.1775G > A p. (Gly592Glu) (line 4), c.2483delA p. (Tyr828Phefs∗101) (line 5), c.661G > T p. (Asp221Tyr) (line 6), c.1216C > T p. (Arg406Trp) (line 7), c.2177C > T p. (Thr726Ile) (line 8), Hek293T-*ldlr*G1 non-transfected (line 9), respectively. The 25 kDa signals represent the DsRed2 protein, indicating the correct transfection process. The 50 kDa bands demonstrate β-actin. **B:** LDLR signals were normalized to the intensity of DsRed2. The relative level of LDLR variants was assessed using the ratio of the variants compared to wild-type band intensity. The mean of four independent Western blots (n = 4) is shown in the graph. The green line is the 90 % threshold, and the red line is the 70 % threshold. Asterisks indicate statistically significant results in the T-test.

The cellular LDLR variant expression in lysates of transfected HEK293T-*ldlr*G1 cells was measured using Western blotting by equal loading of total protein onto a gel and normalizing the obtained signals to DsRed2, which informed about the transfection efficiency. The anti-LDLR antibody indicated the mature form of the receptor at 150 KDa. The band intensity of examined LDLR alterations was reduced compared to the wild-type and was similar to the band intensity of pathogenic variants. The signal of the c.2177C > T p. (Thr726Ile) variant corresponded to the native LDLR expression (Fig. 1; Supplementary S2).

**Fig. 2:**
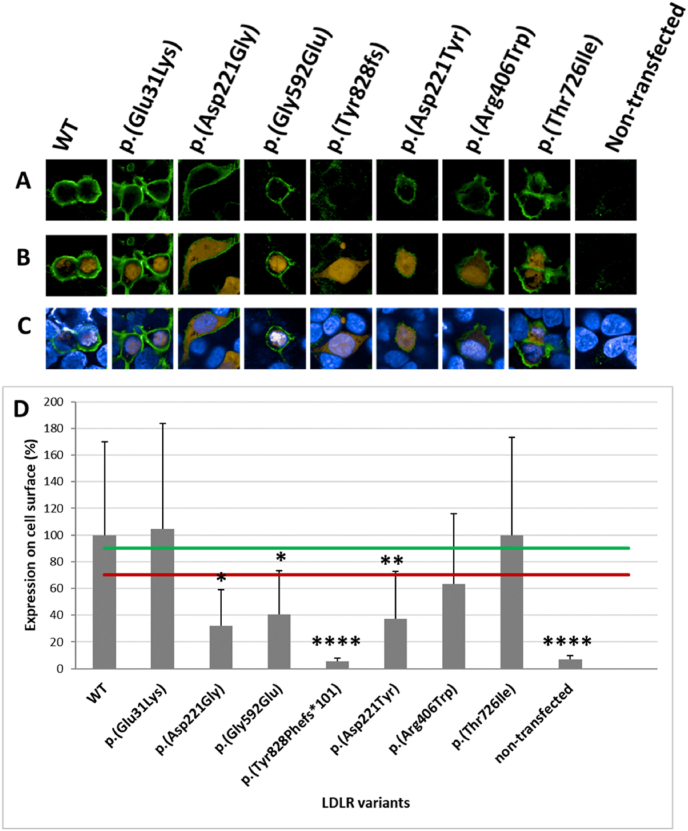
Analysis of LDLR variant expression on the cell membrane by confocal microscope. **A-C:** Transfected cells immunostained with anti-LDLR and Alexa Fluor™ 488 goat anti-mouse IgG conjugated antibodies. The green fluorescence intensity indicated the receptor expression level on a cellular surface. The yellow fluorescence of DsRed2 monitored transfected cells. The nucleus stained with PureBlu™ Hoechst 33342 was visible as blue fluorescence. **D:** The percentage of LDLR was represented on the graph as the mean ± SD (n = 30 cells). The green line is the 90 % threshold, and the red line is the 70 % threshold. Asterisks indicate statistically significant results in the T-test.

The cell membrane LDLR variant expression was investigated by immunostaining receptors using a primary anti-LDLR antibody and secondary Alexa Fluor™ 488 goat anti-mouse IgG conjugated antibody. Observed in the confocal microscope, the yellow fluorescence of DsRed2 remained constant, while the green fluorescence intensity of the receptor was dependent on protein expression and calculated using ImageJ software. The measurement indicated lower expression of c.662A > G p. (Asp221Gly), c.1775G > A p. (Gly592Glu), c.2483delA p. (Tyr828Phefs∗101), c.661G > T p. (Asp221Tyr) and c.1216C > T p. (Arg406Trp) variants. However, the expression of c.91G > A p. (Glu31Lys) and c.2177C > T p. (Thr726Ile) variants were comparable to the expression of native LDLR on a cellular surface (Fig. 2).

**Fig. 3:**
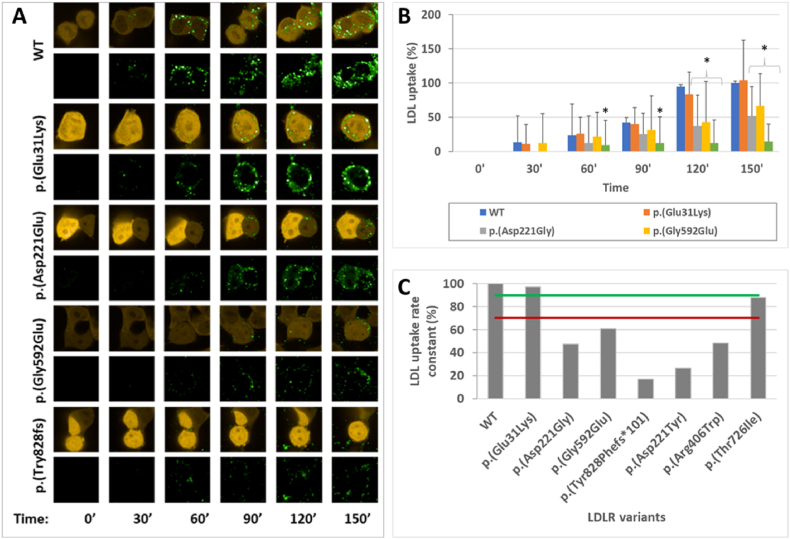
Internalization capacity of LDLR variants. **A:** HEK293T-*ldlr*G1 cells transfected with pTetRedLDLR expression vectors were incubated with the pHrodo™ Green-LDL. LDL uptake was observed in a confocal microscope as increasing green fluorescence over time. Yellow fluorescence of DsRed2 indicated transfected cells. **B:** The increment of internalization capacity of LDLR variants over time is presented as the percentage mean ± SD (n = 50 cells) compared to WT. Asterisks indicate statistically significant results in the T-test. **C:** LDL uptake rate constant. The green line is the 90 % threshold, and the red line is the 70 % threshold.

Variant internalization was assessed as the pHrodo Green-LDL uptake rate constant in a confocal microscope and calculated using ImageJ software. The yellow fluorescence of DsRed2 was persistent and monitored the transfected cells during the experiment. The green fluorescence of pHrodo™ Green-LDL was increasing over time inside the cells. The uptake rate constant of c.91G > A p. (Glu31Lys) and c.2177C > T p. (Thr726Ile) variants reached a cut-off value of approximately 90 %. However, the internalization capacities of variants c.662A > G p. (Asp221Gly), c.1775G > A p. (Gly592Glu), c.2483delA p. (Tyr828Phefs∗101), c.661G > T p. (Asp221Tyr) and c.1216C > T p. (Arg406Trp) were below the 70 % threshold (Fig. 3).

**Fig. 4:**
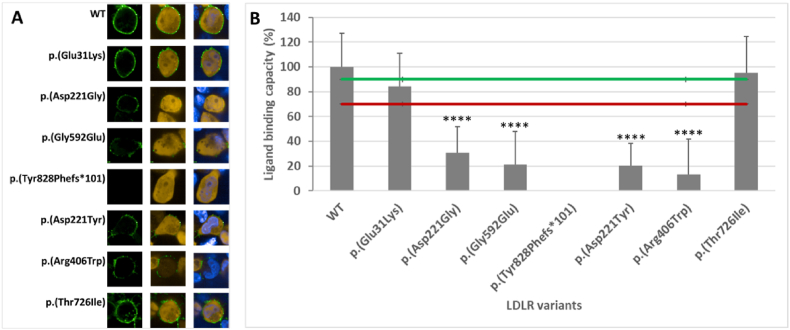
Measurement of ligand binding capacity by LDLR variants. **A:** HEK293T-*ldlr*G1 cells transfected with pTetRedLDLR expression vector were fixed and incubated with BODIPY™ FL-LDL. The LDL binding to receptors was observed in a confocal microscope as the intensity of green fluorescence. The yellow fluorescence of DsRed2 monitored transfected cells. The nucleus stained with PureBlu™ Hoechst 33342 was visible as blue fluorescence. **B:** Ligand binding capacity represents the percentage mean ± SD (n = 65 cells). Asterisks indicate statistically significant results (p < 0.05) in the T-test. The green line is the 90 % threshold, and the red line is the 70 % threshold.

Ligand binding capacity was measured as the ability of the receptors to bind BODIPY™ FL LDL particles. The endocytosis was stopped by receptor fixation with paraformaldehyde on the cellular surface of transfected cells. Receptors affinity for LDL was determined as the green fluorescence intensity of BODIPY™ FL LDL observed in confocal microscopy and analysis with ImageJ software. The yellow fluorescence of DsRed2 indicated the transfected cells. The LDL affinity of the c.2177C > T p. (Thr726Ile) variant corresponded with the wild-type. The ligand binding capacity of c.91G > A p. (Glu31Lys) substitution ranged from 70 % to 90 %. Cells transfected with variants c.662A > G p. (Asp221Gly), c.1775G > A p. (Gly592Glu), c.661G > T p. (Asp221Tyr), and c.1216C > T p. (Arg406Trp) presented lower affinity, below 70 % threshold, for LDL. Moreover, the affinity of the c.2483delA p. (Tyr828Phefs∗101) variant to LDL was undetectable (Fig. 4).

*In silico* analyses based on publicly available packages assessed the deleterious effects of gene alterations on the protein, except for the c.2177C > T p. (Thr726Ile) variant, which was estimated to be benign. The results are presented in Table 1.

**Table 1:** Key features of the variants under study, their ACMG/VCEP classification prior to the functional studies, and the number of probands in the analyzed cohort.

No	cDNA^a^	Protein	Protein Domain^b^	ClinVar ID	ClinGen Allele Registry	ClinGen Expert Panel FH VCEP	functional evidence PS3/BS3Criterion^c^	Allele Frequency^d^	*In silico* analysis		N probands
									REVEL	MES	
1	c.91G > A	p. (Glu31Lys)	LDL-receptor class A1	251013	CA41752	–	–	0.00001460	0.777	<thresholds	3
2	c.662A > G	p. (Asp221Gly)	LDL-receptor class A5	183092	CA023739	PATHOGENIC PP1_strong, PS3_moderate, PS4, PM1, PM2, PM3, PM5,PP3, PP4, BS4	PS3_Moderate (Level 2 assay)	0.00002629	0.986	<thresholds	86
3	c.1775G > A	p. (Gly592Glu)	LDL-receptor class B5	161271	CA023577	PATHOGENIC PS4, PP1_Strong, PM2, PS3_Moderate, PP3, PP4	PS3_Moderate (Level 2 assay	0.00004786	0.938	<thresholds	246
4	c.2483delA	p. (Tyr828Phefs∗101)	NPXY motif	–	–	–	–	–	–	<thresholds	1
5	c.661G > T	p. (Asp221Tyr)	LDL-receptor class A5	251356	CA10585046	PATHOGENIC PP1_Strong, PS3, PM1, PM2, PS4_Moderate, PP3, PP4	PS3 (Level 1 assay)	–	0.978	<thresholds	0
6	c.1216C > T	p. (Arg406Trp)	LDL-receptor class B1	226351	CA033073	PATHOGENIC PM2, PP3, PS3, PS4, PP1_Strong, PP4	PS3 (Level 1 assay)	0.00002004	0.883	<thresholds	0
7	c.2177C > T	p. (Thr726Ile)	Clustered O-linked oligosaccharides	36461	CA023649	BENIGN BA1, BP4	–	0.008325	0.454	<thresholds	0

a NM_000527.2.

b UniProt database.

c ClinGen Familial Hypercholesterolemia Expert Panel Specifications to the ACMG/AMP Variant Classification Guidelines.

d GnomAD v4.1.0, Grpmax Filtering AF, Exomes.

The data obtained from molecular studies, based on the *in vitro* tool HEK293T-*ldlr*G1pTetRedLDLR, allowed to use PS3 as strong level in classification of c.662A > G p. (Asp221Gly), c.1775G > A p. (Gly592Glu), and c.2483delA p. (Tyr828Phefs∗101) variants (Table 2).

**Table 2:** *In vitro* analysis of LDLR variants.

No	cDNA^a^	Protein	Cellular Expression	Cell Surface Expression	LDL Uptake	LDL Binding	PS3 criterion^b^ assay result of <70 % of wild-type activity in either expression/biosynthesis, binding OR internalization	BS3 criterion^b^ assay result >90 % of wild-type activity in expression/biosynthesis, binding AND internalization.
			Percentage (%)					
1	c.91G > A	p. (Glu31Lys)	0.1	104.8	89.3	84.1	NO	NO
2	c.662A > G	p. (Asp221Gly)	47.7	32.2	47.4	30.7	YES	NO
3	c.1775G > A	p. (Gly592Glu)	7.4	40.6	37.1	21.1	YES	NO
4	c.2483delA	p. (Tyr828Phefs∗101)	0.1	5.5	16.8	0	YES	NO
5	c.661G > T	p. (Asp221Tyr)	38.6	37.5	26.7	20.3	YES	NO
6	c.1216C > T	p. (Arg406Trp)	22.2	63.3	48.3	13.2	YES	NO
7	c.2177C > T	p. (Thr726Ile)	83.7	100.0	87.8	95.3	NO	NO

a NM_000527.2.

b ClinGen Familial Hypercholesterolemia Expert Panel Specifications to the ACMG/AMP Variant Classification Guidelines.

## 4 Discussion

In total, 3995 probands were screened for germline alterations in primary genes associated with familial hypercholesterolemia. A molecular analysis was performed from January 2002 to January 2025. A study of the above cohort identified several hundreds of *LDLR* variants, which had to be classified for clinical purposes. Variants have been classified/reclassified according to ACMG/AMP guidelines [12] since 2015 and gene-specific recommendations established by the ClinGen FH Variant Curation Expert Panel (VCEP) since 2021.

In this study, two common missense *LDLR* variants were selected to analyze their impact on LDL receptor function. Variants c.1775G > A p. (Gly592Glu) and c.662A > G p. (Asp221Gly) have already been classified by ClinGen Experts; however, the data from the level 1 assay have not yet been published. Most published results from functional analysis *in vitro* and/or *in vivo* for the above variants are in agreement. However, Thormaehlen et al. classified the c.1775G > A p. (Gly592Glu) variant as “non-disruptive” based on LDL cholesterol level and as “benign” based on the results of functional studies [25]. Those data are not included or commented on in the VCEP classification record. However, in the case of other variants which were classified as “benign” by authors, ClinGen Experts reject the criterion BS3 based on those results, except for variant c.1720C > T (p. (Arg574Cys), where the data were recognized as BS3 supporting criterion.

The variant c.91G > A p. (Glu31Lys) was also examined. It was described as a novel variant in Polish and Dutch cohorts of hypercholesteremic children [17,26] Initially, it was categorized as a nonpathogenic variant based on PolyPhen and SIFT prediction. However, it was detected in other hypercholesteremic patients in the following years, including single informative meiosis in the Dutch family [27]. Moreover, two additional FH probands were diagnosed at our Center based on validated clinical criteria, having 6 and 8 Dutch Lipid Clinic Network points [unpublished data]. According to the data available during the preparation of the manuscript, the c.91G > A p. (Glu31Lys) variant was not classified by ClinGen Experts. It was annotated as pathogenic in the LOVD database and inconclusive in the ClinVar database: likely pathogenic (1x), uncertain significance (5x) (ID: 251013). Based on ClinGen gene-specific guidelines, it was classified as VUS in our Centre by applying PM2, PP3, and PP4 evidence codes. The presented data is insufficient to classify the c.91G > A p. (Glu31Lys) variant as pathogenic or benign, but it shows that the variant does not act as a loss of function LDLR mutation. However, the BS3 criterium is not fulfilled because the assay result did not show >90 % of wild-type protein activity in the expression/biosynthesis of the variant with the full LDL binding and internalization An equivocal expression result of c.91G > A p. (Glu31Lys) variant may be due to the substitution in the LDL-receptor class A1 domain which is the epitope recognized by the primary anti-LDLR antibody. Unfortunately, the use of other primary anti-LDLR antibodies recognizing an epitope in a different region of the LDLR lead to cross-reaction with other cellular components, giving a false result [unpublished data].

Stop-loss and frameshift variants extending translation beyond the reference stop codon into the 3′ untranslated region are predicted to generate extended proteins. Frameshift variants extending the sequence also disrupt the original amino acid sequence, which may impact functionally relevant regions. The prevalence, mechanism of pathogenicity, and clinical impact of these variants are not well characterized, which presents a challenge for variant classification. c.2483delA p. (Tyr828Phefs∗101) variant is located in the penultimate exon of the *LDLR* gene and affects the last 33 amino acids of LDLR (<10 % of protein), leading to protein extension beyond the regular stop codon. Variants leading to premature protein termination and loss of essential functional domains are typically classified as pathogenic. However, the impact of frameshift variants resulting in an extended incorrect terminus is unclear. The eukaryotic cells have translational control pathways that repress the activity of deleterious mRNAs. While transcripts with a premature stop codon undergo nonsense-mediated decay, elongated transcripts can be degraded via nonstop decay. The Tayoun et al., 2018 PVS1 decision tree, nor VCEP adaptation, does not show a straightforward option in this situation. We show that the c.2483delA p. (Tyr828Phefs∗101) variant with a lack of expression and undetectable ligand binding can be described as a null variant.

This study aimed to perform an experimental analysis of the two most common substitutions of the *LDLR* gene, c.1775G > A p. (Gly592Glu) and c.662A > G p. (Asp221Gly) as well as c.91G > A p. (Glu31Lys) and c.2483delA p. (Tyr828Phefs∗101) stop loss alteration detected in the Polish population to reclassify the variants, which is essential for the correct diagnosis of FH patients. The obtained data were compared with the wild-type and control variants, pathogenic (c.661G > T p. (Asp221Tyr) and c.1216C > T p. (Arg406Trp)) and benign (c.2177C > T p. (Thr726Ile)) [[18], [19], [20]].

The functional analysis of the selected *LDLR* variants was performed using the *LDLR* knock-out cell line HEK293T-*ldlr*G1. The HEK293T-*ldlr*G1 cells are the second LDLR-deficient cell line to be described after the CHO-*ldl*A7 cell line [28]. The cells were established by CRISPR/Cas9-mediated luciferase-puromycin knock-in and are new valuable tools for studying FH [29]. The cells were transfected by bicistronic expression vectors carrying the *LDLR* gene variants and reporter gene *DsRed2* under the control of the P_TRE3G_ promoter. The level of LDLR and DsRed2 expression depends on doxycycline concentration in the medium, and it could be tuned according to the requirements of experiments. Moreover, the red fluorescence of DsRed2 helps monitor transfected cells during confocal microscopy assays. The validity of our functional analysis model has been assessed and described previously [16].

The data collected during the molecular analysis revealed that the most common substitutions c.1775G > A p. (Gly592Glu) and c.662A > G p. (Asp221Gly) detected in the Polish cohort are disease-causing due to reduced expression, ligand binding, and internalization defects. Their abolished function was comparable to the pathogenic control variants.

The internalization and LDL binding capacity of c.91G > A p. (Glu31Lys) variant reached approximately 90 % of the wild-type and benign control variants. However, the expression level was reduced due to the substitution found in the LDLR class A1 domain, which interacts with anti-LDLR antibody. The c.91G > A p. (Glu31Lys) variant was assessed with likely regular activity.

The present work facilitated a better understanding of the mechanism of FH development and the impact of genetic alterations on receptor activity. Furthermore, the obtained results will contribute to the correct classification of variants, thus enabling the correct diagnosis and optimal treatment of FH patients.

## Author contributions

“Conceptualization, B.W. and J.J.; methodology, J.J., M.T., A.JJ., M.C., M.Ż., L.K., K.W. and B.W.; validation, J.J., M.T., A.JJ., M.C., M.Ż., K.C., M.G., L.K., K.W. and B.W.; formal analysis, J.J., M.T., A.JJ., M.C., M.Ż., L.K., K.W. and B.W.; investigation, J.J., M.T., A.JJ., and B.W.; resources, J.J., L.K., K.W. and B.W.; writing—original draft preparation M.T.,M.C, J.J and B.W.; visualization, M.T., J.J., A.JJ., L.K., K.W., B.W.; supervision, J.J. and B.W.; project administration, J.J, B.W.; funding acquisition, B.W. All authors have read and agreed to the published version of the manuscript.

## Financial support

This work was supported by NCN, grant no. 10.13039/100025296OPUS 2015/19/B/NZ5/03510, and by the Ministry of Education and Science Poland, grant no. 2/566516/SPUB/SP/2023.

## Declaration of competing interest

The authors declare that they have no known competing financial interests or personal relationships that could have appeared to influence the work reported in this paper.

## Data Availability

Available on request through collaborations.

## Abbreviations

CAD, coronary artery disease; FH, familial hypercholesterolemia; LDL, low-density lipoprotein; LDL-C, low-density lipoprotein cholesterol; LDLR, LDL receptor; MES, maximum entropy scan; REVEL, rare exome variant ensemble learner; SD, standard deviation; VCEP, ClinGen FH Variant Curation Expert Panel; VUA, variant under assessment; WT, wild type.
