# Supplementary material for: Characterization of selected LDLR substitutions in patients with familial hypercholesterolemia
Source: Atheroscler Plus. 2025 Nov 18;62:30–7. doi: 10.1016/j.athplu.2025.11.001 (PMC12681971; doi:10.1016/j.athplu.2025.11.001)
Supplement: Multimedia component 1 [file mmc1.docx]

**Supplementary S1 Primer and template list pTetRedLDLR expression vectors**

**Template:**

- Expression vector pIRES2-DsRED (Takara Bio Inc., Shiga, Japan)
- Expression vector pCMV6-LDLR (NM_000527.2; OriGene; MD, USA)
- mRNA was isolated from the HUVEC cell line Catalog #: C2519A (Lonza; Basel, Switzerland)

**Primers:**

**pIRES2dsRED – PCR fragment 1- dsRed2 - 708 bp with:**

DsRed Forward: 5’- ACCCTCGTAAAGAATTCACCATGGCCTCCTCCGAGAA

DsRed Reverse: 5’- GGATCCCGGGCTACAGGAACAGGTGGTGGCG

**pIRES2dsRED – PCR fragment 2- IRES2 - 606 bp with:**

IRES 2 Forward: 5’- TCCTGTAGCCCGGGATCCGCCCCTCTCCCTC

IRES 2 Reverse: 5’- GGTTGTGGCCATATTATCATCGTG

**Wild-type:**

**pCMV6-LDLR – PCR fragment 3- LDLRwt -2627 bp with:**

IRES_N_LDLR: 5’- GATAATATGGCCACAACCGCGAGCAT

GGGGCCCTGGGGC

C-LDLR_Reverse: 5’- CCTCTAGACATATGCTGCAGTCACGCCACGTCAT

CCTCCAG

**1. c.91G>A p.(Glu31Lys),**

**pCMV6-LDLR – PCR fragment 3- LDLR c.91G>A part1 – 115 bp with**

IRES_LDLR_Forward: 5’ – GATAATATGGCCACAACCGCGAGCATGGGG

CCCTGGGGC

p.(Glu31Lys)_Reverse: 5’- TCTTGGCACTGGAACTTGTTTCTTTCGCAT

**pCMV6-LDLR – PCR fragment 4- LDLR c.91G>A part2 - 2512 bp with**

p.(Glu31Lys)_ Forward: 5’- ATGCGAAAGAAACAAGTTCCAGTGCCAAGA

C_LDLR_Reverse: 5’- CCTCTAGACATATGCTGCAGTCACGCCACGTCAT

CCTCCAG

**2. c.662A>G p.(Asp221Gly)**

**pCMV6-LDLR – PCR fragment 3- LDLR c.662A>G part1 -686 bp with**

IRES_LDLR_Forward: 5’- GATAATATGGCCACAACCGCGAGCATGGGGCCCT

GGGGC

p.(Asp221Gly)_Reverse: 5’- GATTTGTCCTTGCAGCCGGGGCCACCATCA

**pCMV6-LDLR – PCR fragment 4- LDLR c.662A>G part2 – 1941 bp with**

p.(Asp221Gly)_ Forward: 5’- TGATGGTGGCCCCGGCTGCAAGGACAAATC

C_LDLR_Reverse: 5’- CCTCTAGACATATGCTGCAGTCACGCCACGTCA

TCCTCCAG

**3. c.1775G>A p.(Gly592Glu)**

**pCMV6-LDLR – PCR fragment 3- LDLR c.1775G>A part1 -1799 bp with**

IRES_LDLR_Forward: 5’- GATAATATGGCCACAACCGCGAGCATGGGGCCCT

GGGGC

p.(Gly592Glu)_Reverse: 5’- TCTTCCGGTTGCCCTCGTTGACATCGATGC

**pCMV6-LDLR – PCR fragment 4- LDLR c.1775G>A part2 - 828 bp with**

p.(Gly592Glu)_ Forward: 5’- GCATCGATGTCAACGAGGGCAACCGGAAGA

C_LDLR_Reverse: 5’- CCTCTAGACATATGCTGCAGTCACGCCACGTCAT

CCTCCAG

**4. c.2483delA p.(Tyr828fs)**

**pCMV6-LDLR – PCR fragment 3- LDLR c.2483delA part1 – 2504 bp with**

IRES_LDLR_Forward: 5’-GATAATATGGCCACAACCGCGAGCATGGGGCCCT

GGGGC

p.(Tyr828fs)_Reverse: 5’- TCTGTGGTCTTCTGAAGACGGGGTTGTCAA

**mRNA-LDLR (HUVEC cell line) – PCR fragment 4- LDLR c.2483delA part2 - 408 bp with**

p.(Tyr828fs)_ Forward: 5’- TTGACAACCCCGTCTTCAGAAGACCACAGA

C_ LDLR_Y828fs_Cterm _LDLR_Reverse:

5’- CCTCTAGACATATGCTGCAGTTCACGAGGAAAGGAA

GAAACC

**5. c.661 G>T p.(Asp221Tyr)**

**pCMV6-LDLR – PCR fragment 3- LDLR c.661 G>T part1 – 682 bp with**

IRES_LDLR_Forward: 5’-GATAATATGGCCACAACCGCGAGCATGGGGCCCT

GGGGC

p.(Asp221Tyr)_Reverse: 5’- CAGATTTGTCCTTGCAGTAGGGGCCACCATCA

**pCMV6-LDLR – PCR fragment 4- LDLR c.661 G>T part2 – 1945 bp with**

p.(Asp221Tyr)_ Forward: 5’- TGATGGTGGCCCCTACTGCAAGGACAAATCTG

C_LDLR_Reverse: 5’- CCTCTAGACATATGCTGCAGTCACGCCACGTCAT

CCTCCAG

**6. c.1216 C>T p.(Arg406Trp)**

**pCMV6-LDLR – PCR fragment 3- LDLR c.1216 C>T part1 – 1237 bp with**

IRES_LDLR_Forward: 5’-GATAATATGGCCACAACCGCGAGCATGGGGCCCT

GGGGC

p.(Arg406Trp)_Reverse: 5’- CTGACCTCGTGCCAGTTGGTGAAGAAGAGG

**pCMV6-LDLR – PCR fragment 4- LDLR c.1216 C>T part2 -1390 bp with**

p.(Arg406Trp)_ Forward: 5’- CCTCTTCTTCACCAACTGGCACGAGGTCAG

C_LDLR_Reverse: 5’- CCTCTAGACATATGCTGCAGTCACGCCACGTCAT

CCTCCAG

**7. c.2177C>T p.(Thr726Ile)**

**pCMV6-LDLR – PCR fragment 3- LDLR c.2177C>T part1 – 2198 bp with**

IRES_LDLR_Forward: 5’- GATAATATGGCCACAACCGCGAGCATGGGGCCCT

GGGGC

p.(Thr726Ile)_Reverse: 5’- CTTTAGCCTGACGATGGATGTCTCCTGGG

**pCMV6-LDLR – PCR fragment 4- LDLR c.2177C>T part2 – 429 bp with**

p.(Thr726Ile)_ Forward: 5’- AGGAGACATCCATCGTCAGGC

C_LDLR_Reverse: 5’- CCTCTAGACATATGCTGCAGTCACGCCACGTCATCC

TCCAG

**Supplementary S2 Western blot analysis**

**DsRed2 fluorescence observed under fluorescence microscope as transfection efficiency**

**
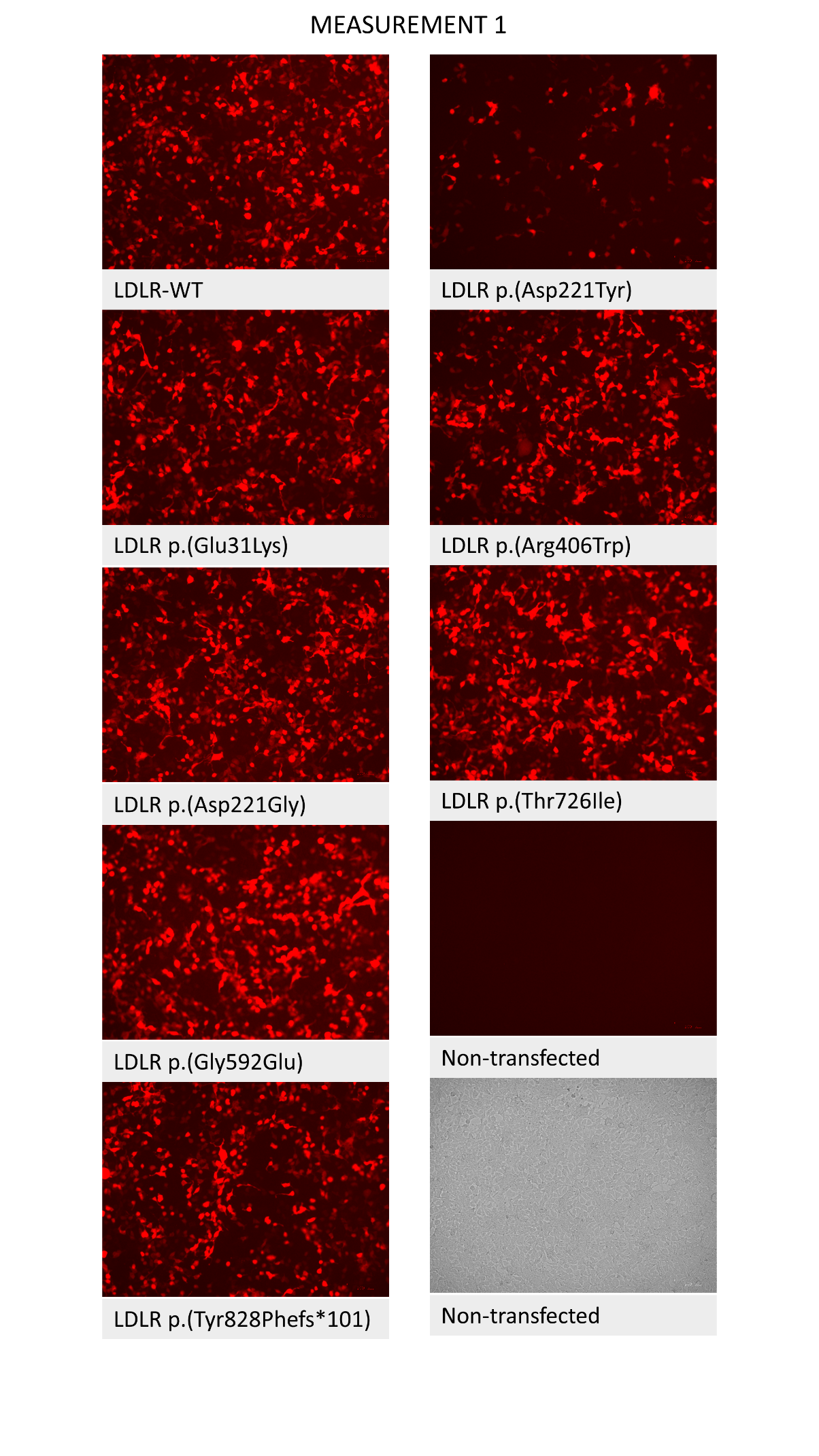
**

**
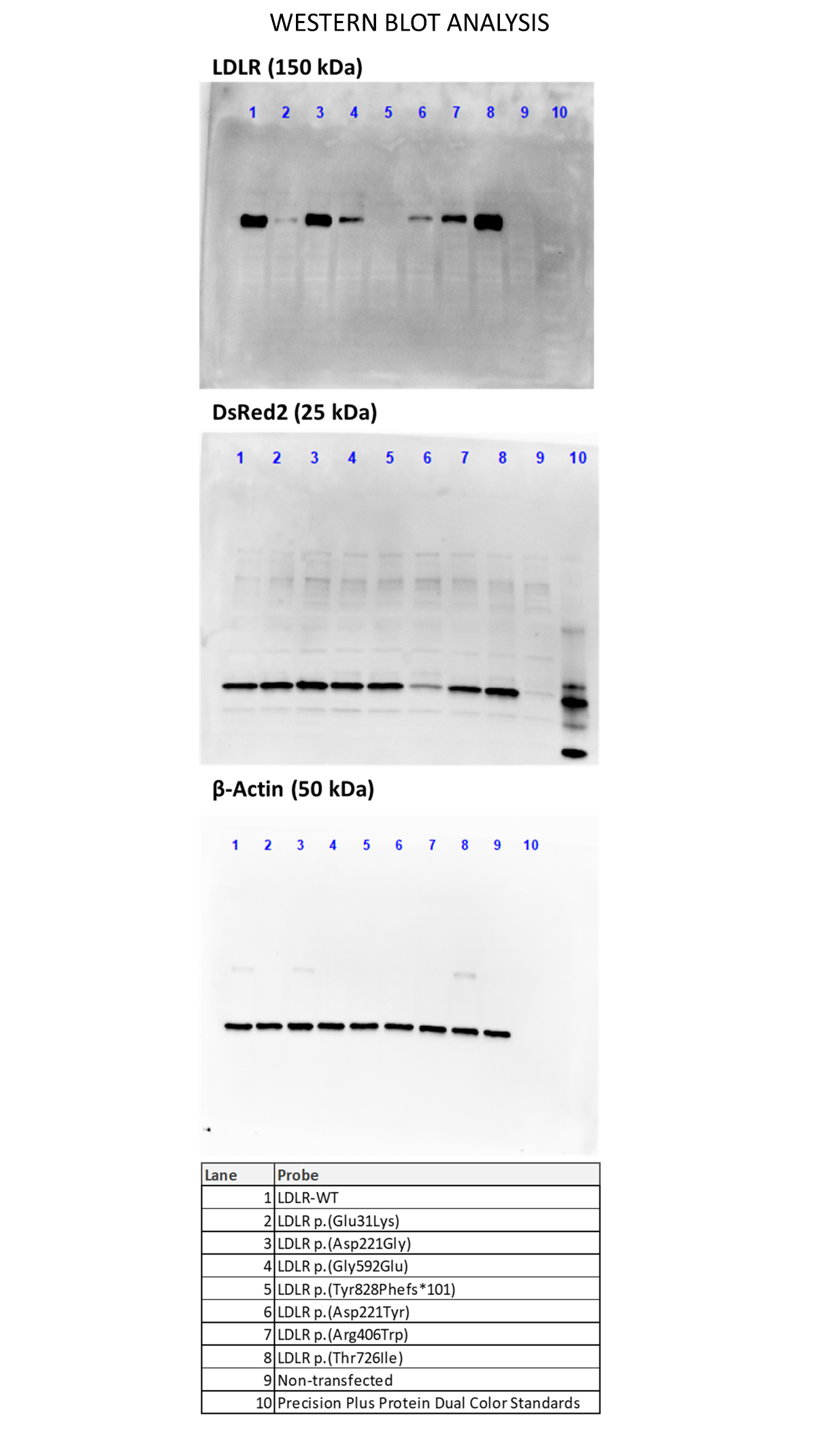
**

**
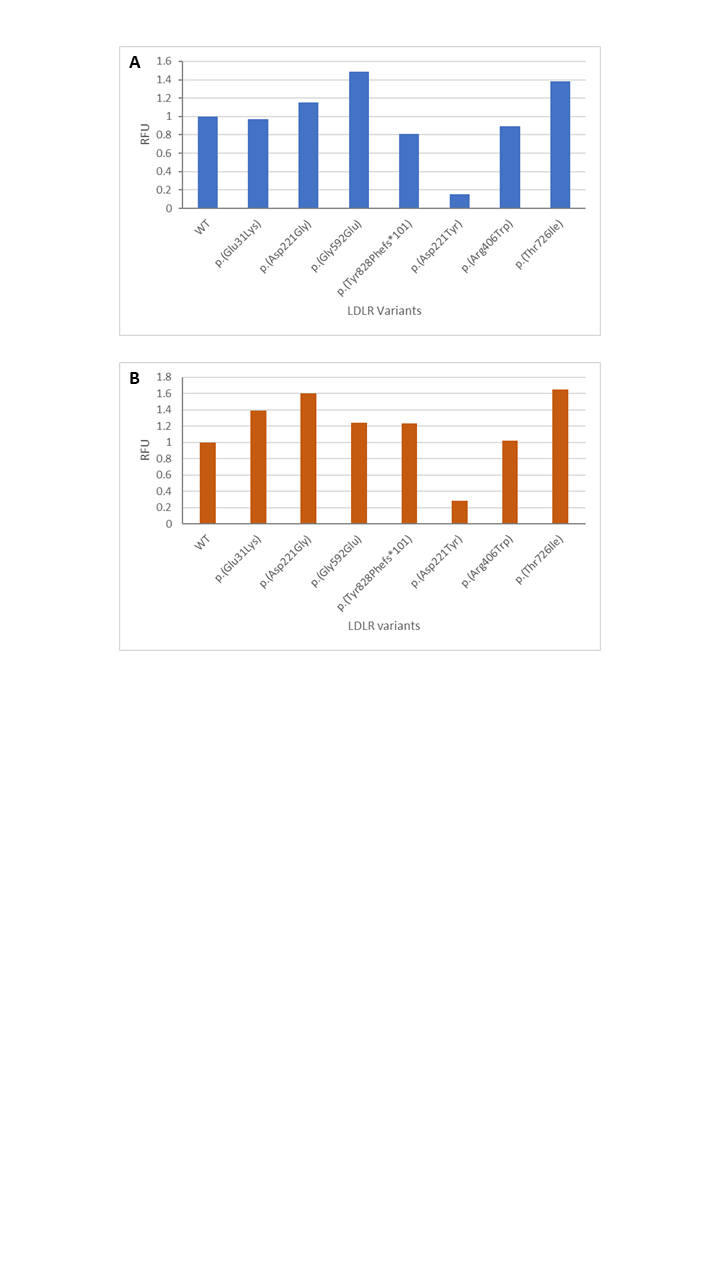
**

**Graph 1 Transfection efficiency measurement 1 (A)** The red fluorescence of DsRed2 was monitored under a fluorescence microscope. Obtained data were calculated using ImageJ software as %Area value^1^ to evaluate the transfection rate. **(B)** The DsRed2 expression in transfected HEK293T-*ldlr*G1 cells was analyzed using a Western blot technique using an anti-DsRed2 primary antibody. The intensity of the bands indicated transfection efficiency and was measured using ImageLab software. LDLR variant expression was normalized to the DsRed2 signal.

^1^ The %Area value is the fraction of the area. For thresholded images, this is the percentage of pixels in the image highlighted in red using the Image-Adjust-Threshold algorithm. Tiago Ferreira, Wayne Rasband; ImageJ User Guide IJ 1.46r

**
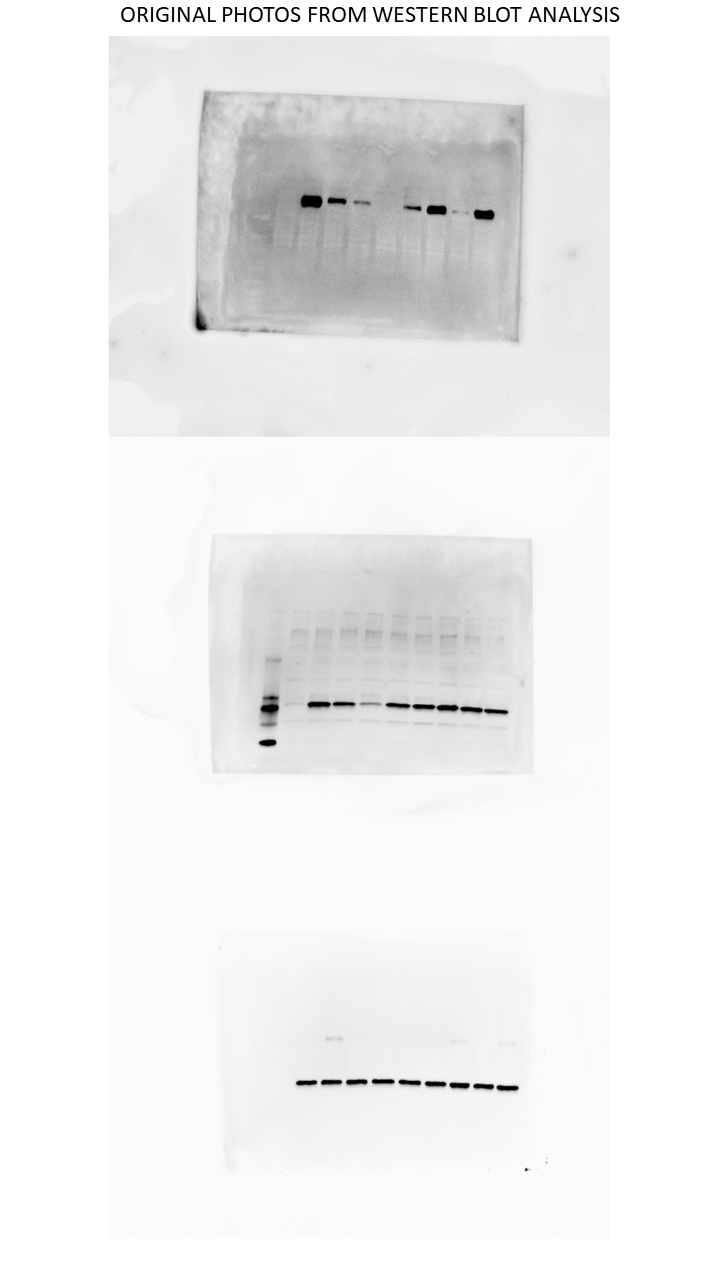
**

**DsRed2 fluorescence observed under fluorescence microscope as transfection efficiency
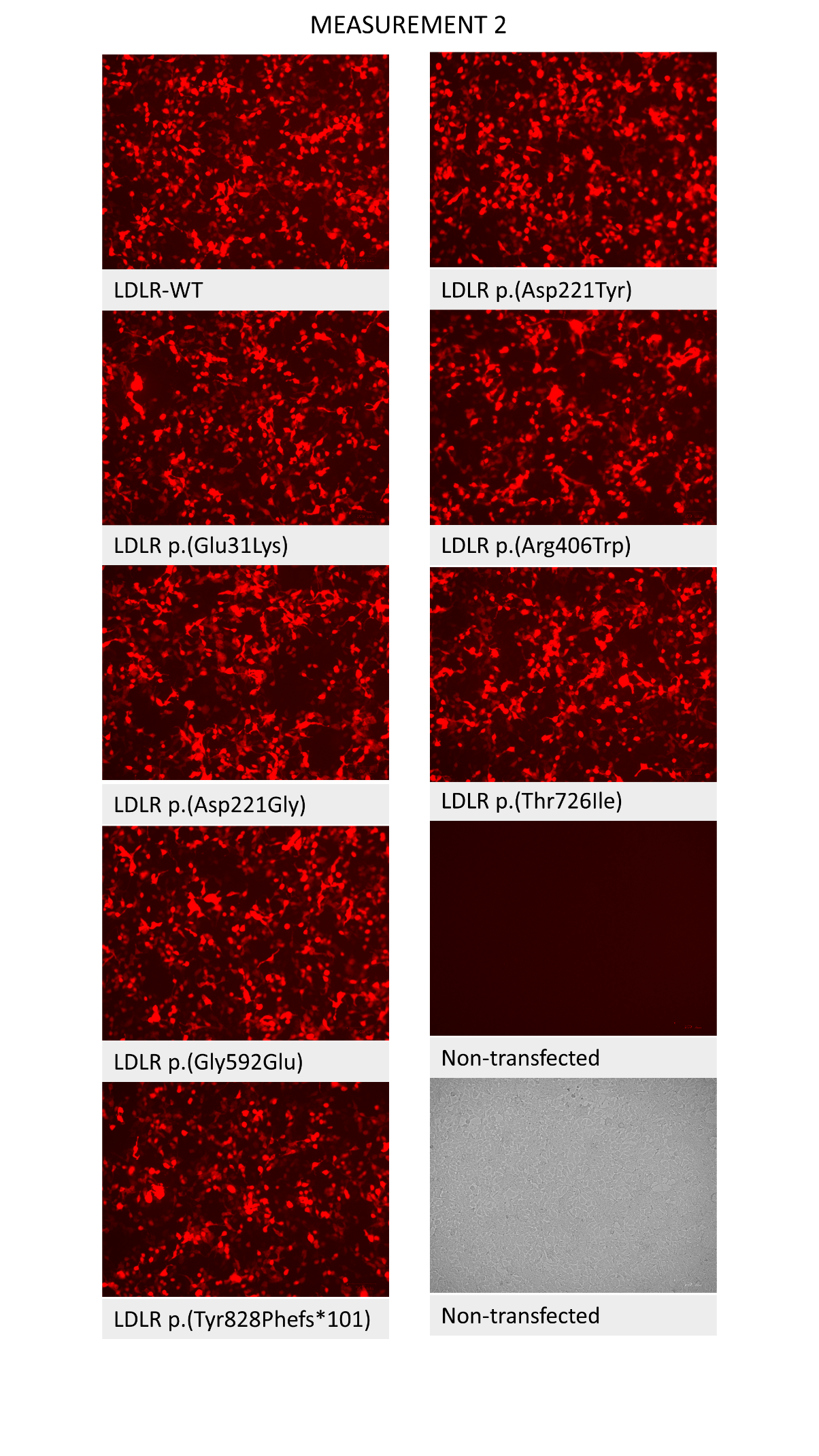
**

**
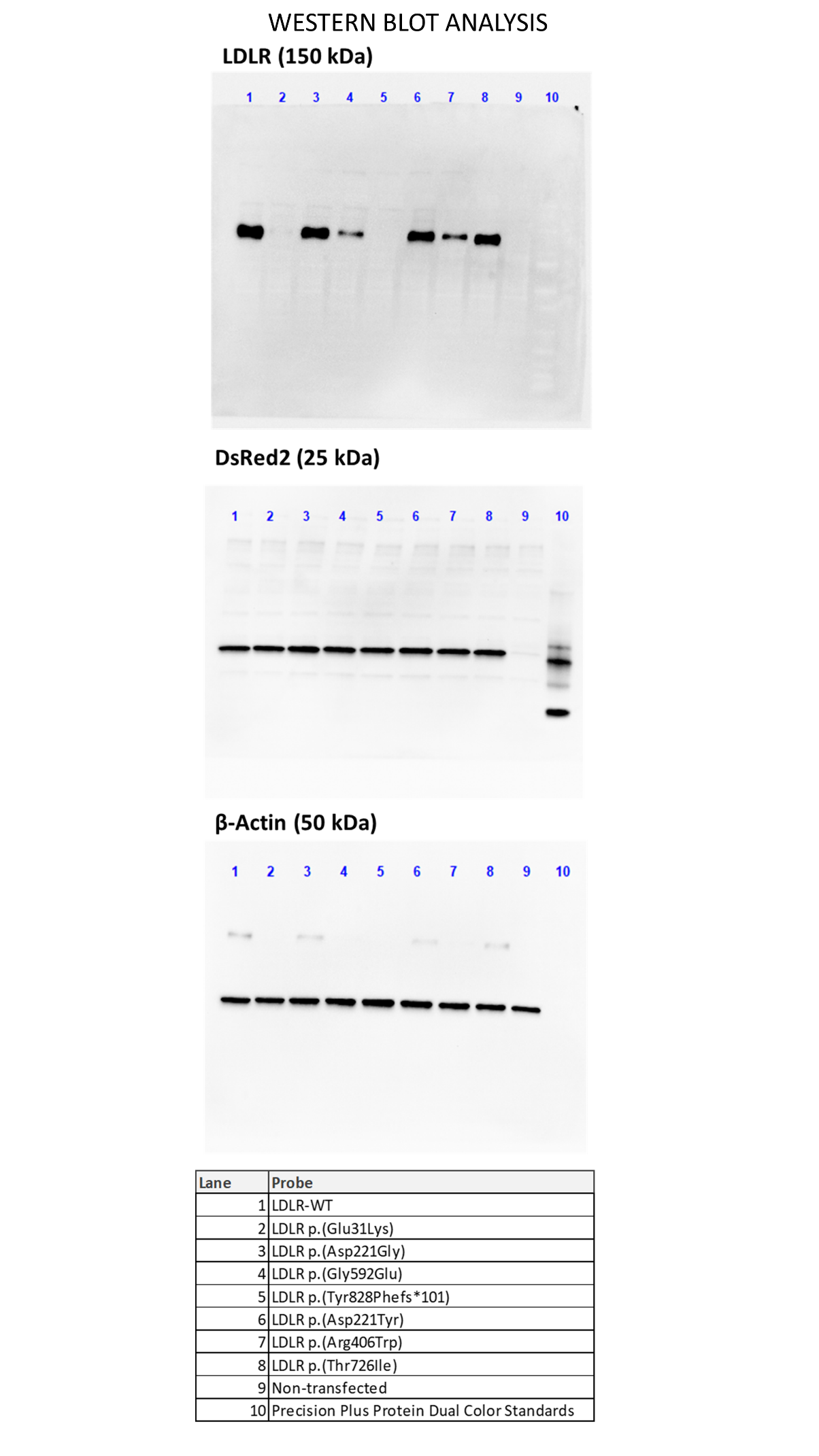
**

**
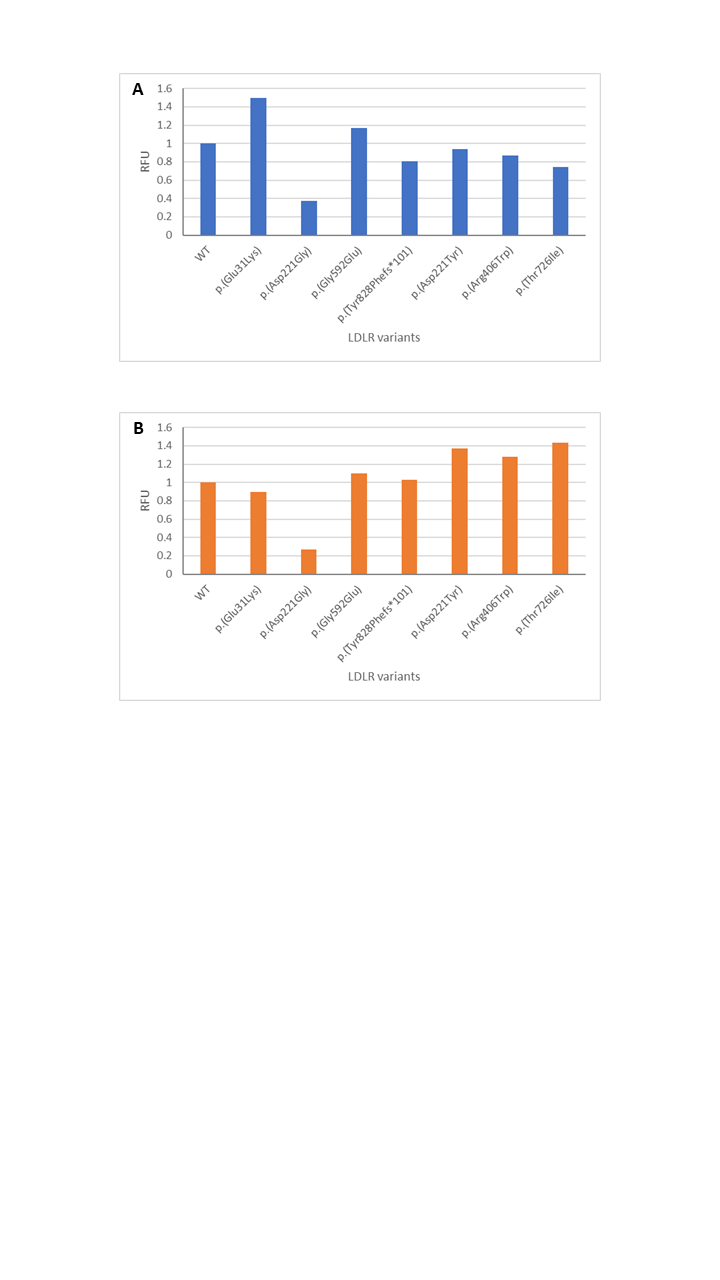
**

**Graph 2 Transfection efficiency measurement 2: (A)** The red fluorescence of DsRed2 was monitored under a fluorescence microscope. Obtained data were calculated using ImageJ software as %Area value^1^ to evaluate the transfection rate. **(B)** The DsRed2 expression in transfected HEK293T-*ldlr*G1 cells was analyzed using a Western blot technique using an anti-DsRed2 primary antibody. The intensity of the bands indicated transfection efficiency and was measured using ImageLab software. LDLR variant expression was normalized to the DsRed2 signal.

^1^ The %Area value is the fraction of the area. For thresholded images, this is the percentage of pixels in the image highlighted in red using the Image-Adjust-Threshold algorithm. Tiago Ferreira, Wayne Rasband; ImageJ User Guide IJ 1.46r

**
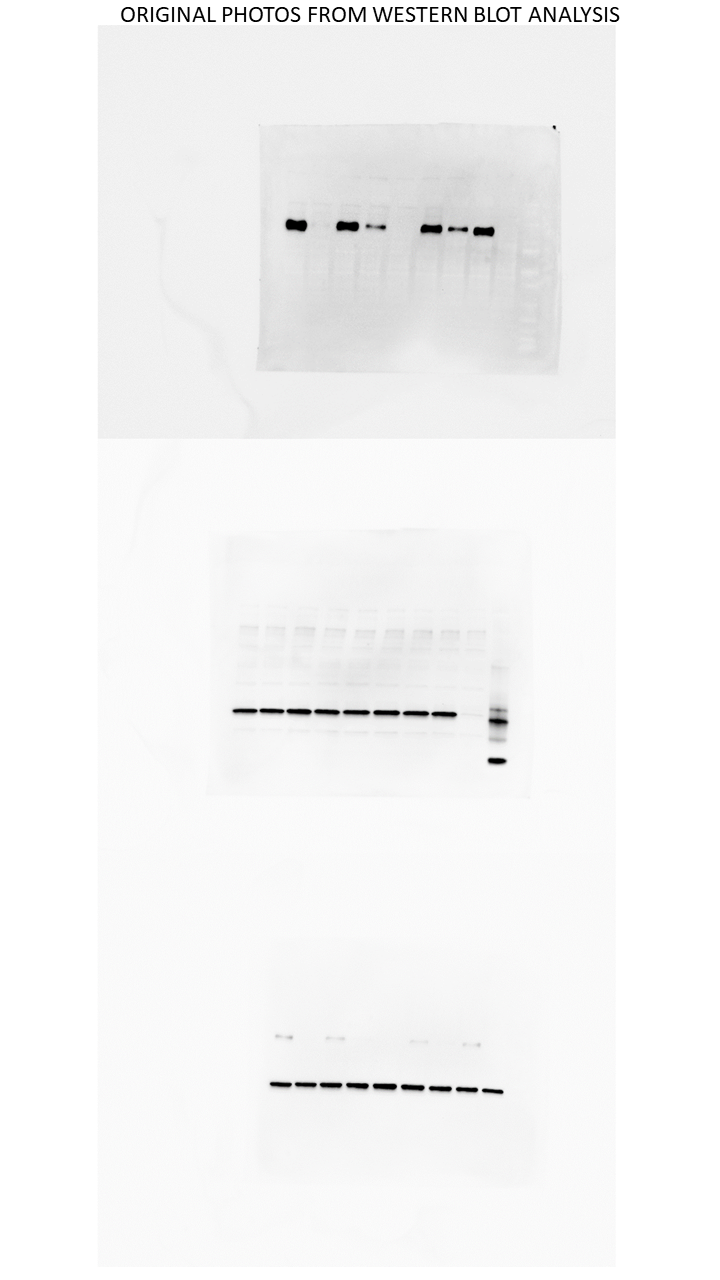
**

**DsRed2 fluorescence observed under fluorescence microscope as transfection efficiency**

**
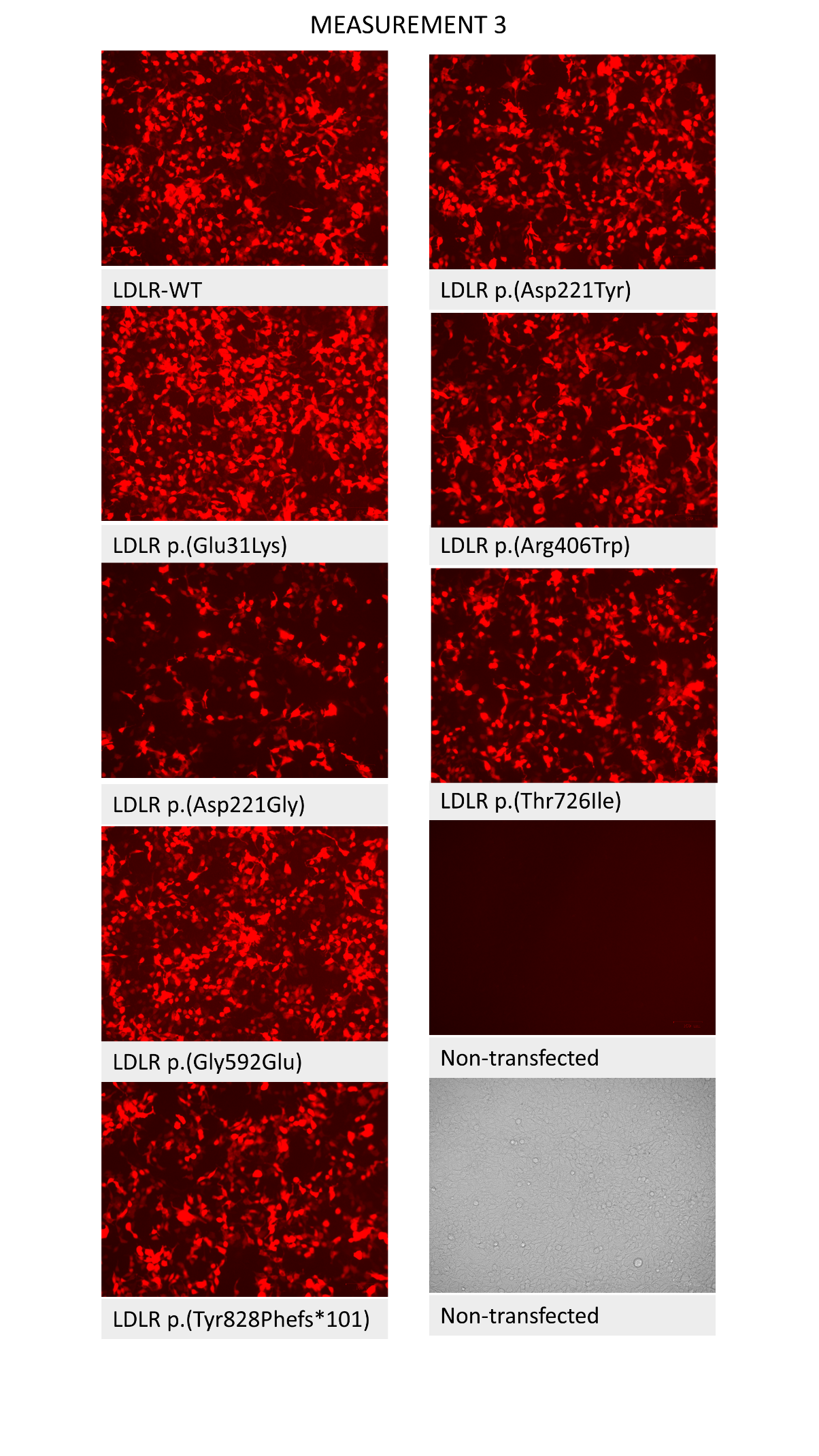
**

**
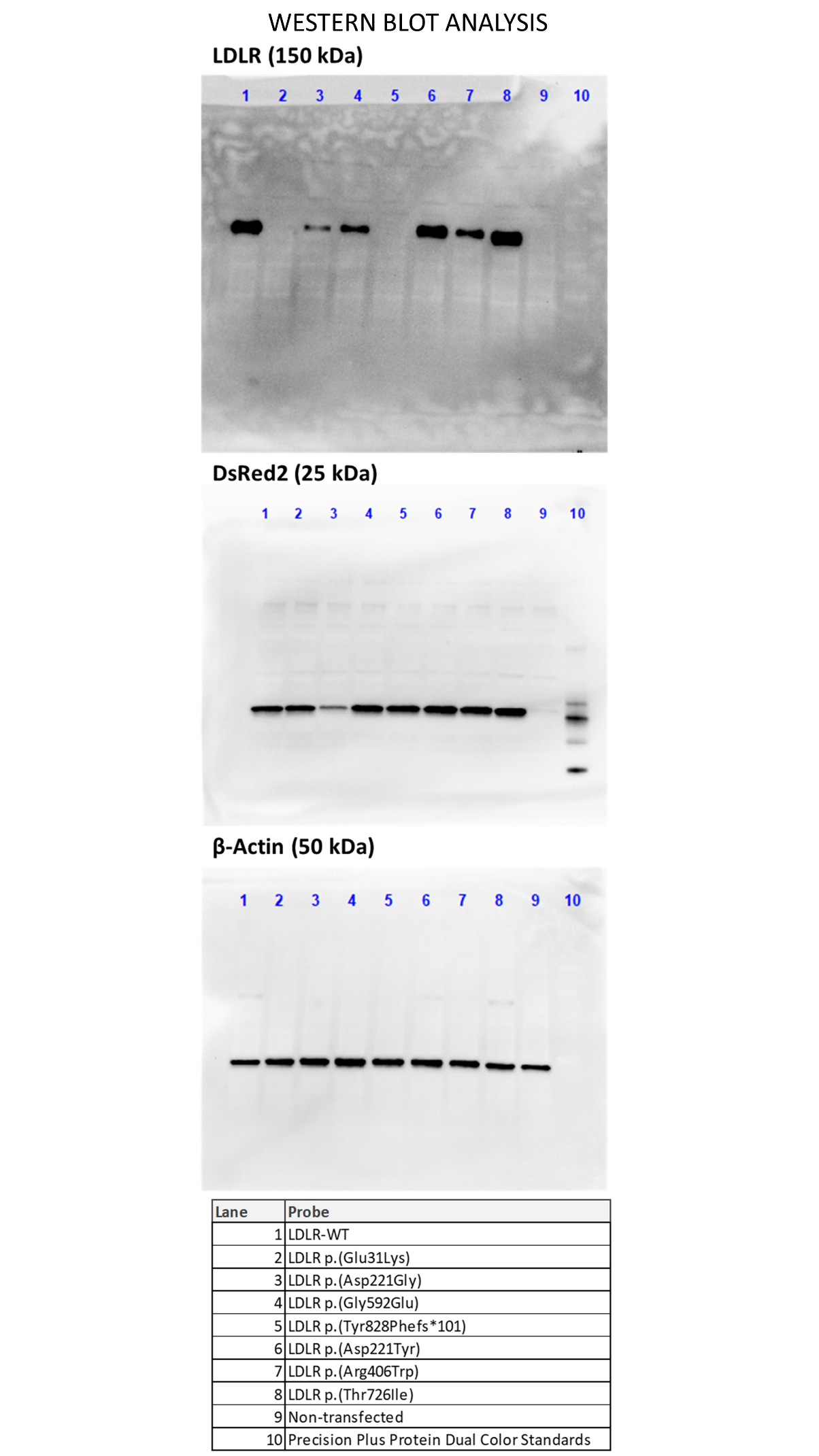
**

**
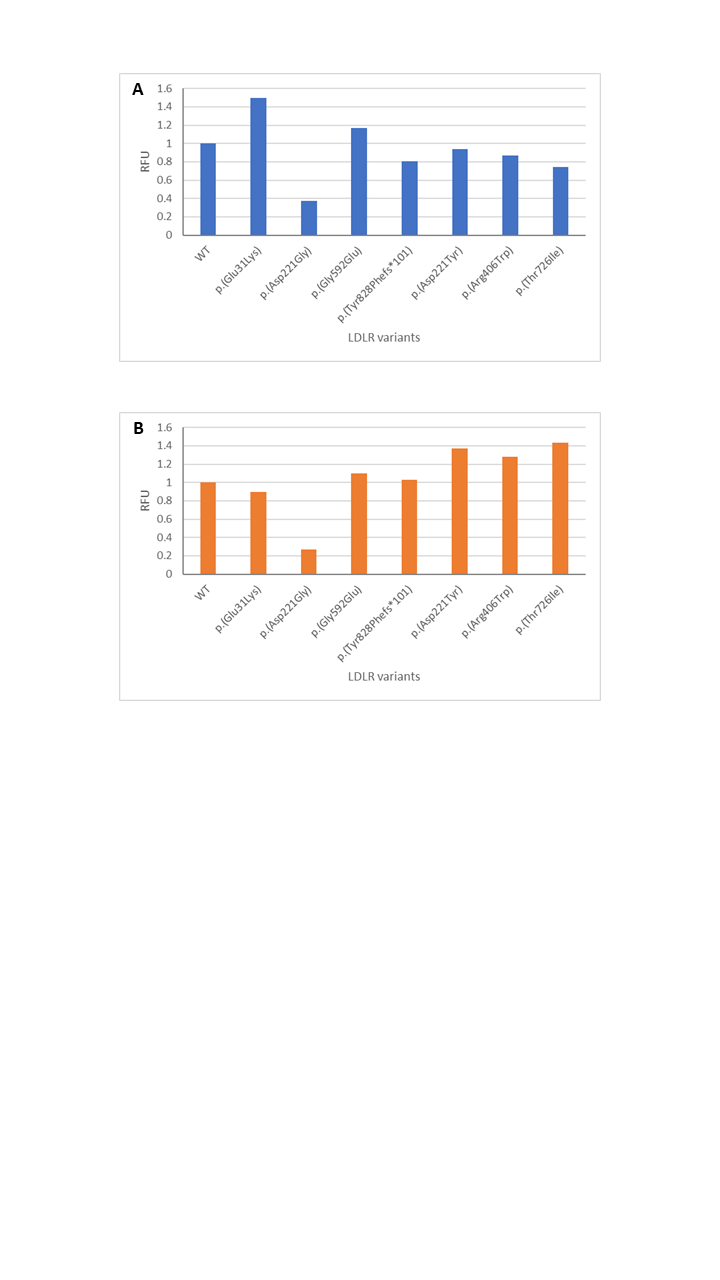
**

**Graph 3 Transfection efficiency measurement 3 (A)** The red fluorescence of DsRed2 was monitored under a fluorescence microscope. Obtained data were calculated using ImageJ software as %Area value^1^ to evaluate the transfection rate. **(B)** The DsRed2 expression in transfected HEK293T-*ldlr*G1 cells was analyzed using a Western blot technique using an anti-DsRed2 primary antibody. The intensity of the bands indicated transfection efficiency and was measured using ImageLab software. LDLR variant expression was normalized to the DsRed2 signal.

^1^ The %Area value is the fraction of the area. For thresholded images, this is the percentage of pixels in the image highlighted in red using the Image-Adjust-Threshold algorithm. Tiago Ferreira, Wayne Rasband; ImageJ User Guide IJ 1.46r

**
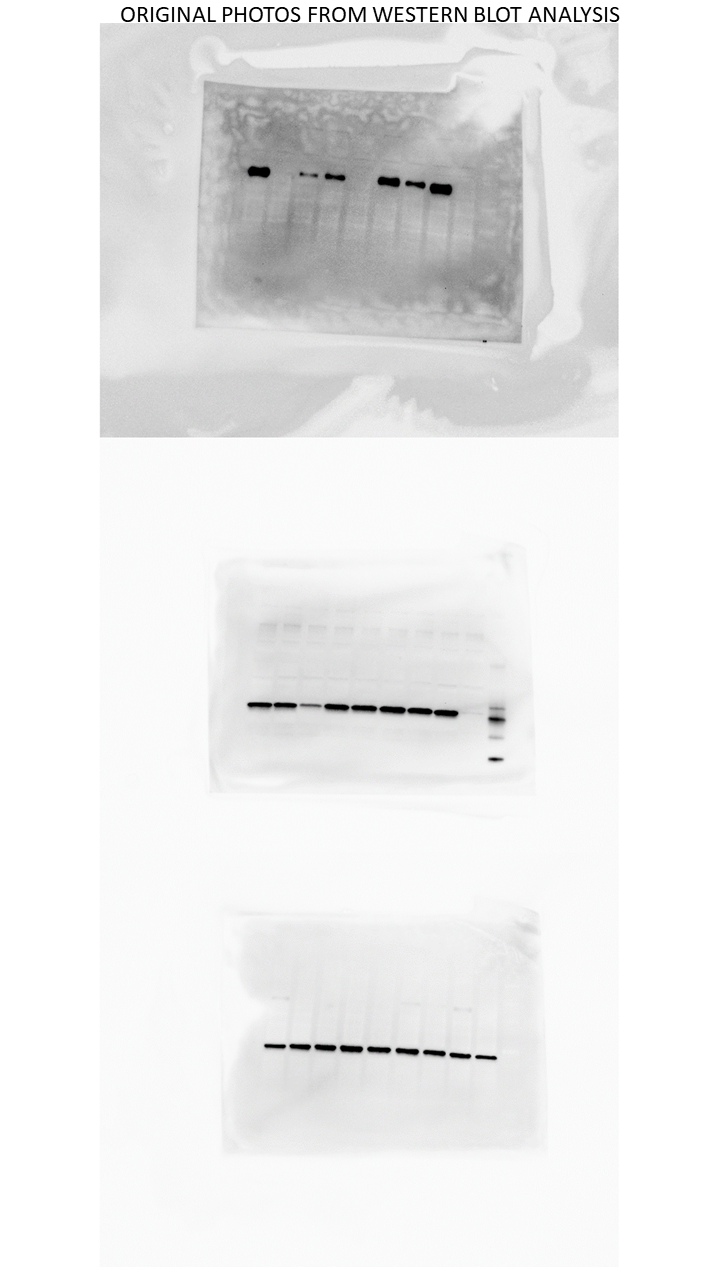
**

**DsRed2 fluorescence observed under fluorescence microscope as transfection efficiency**

**
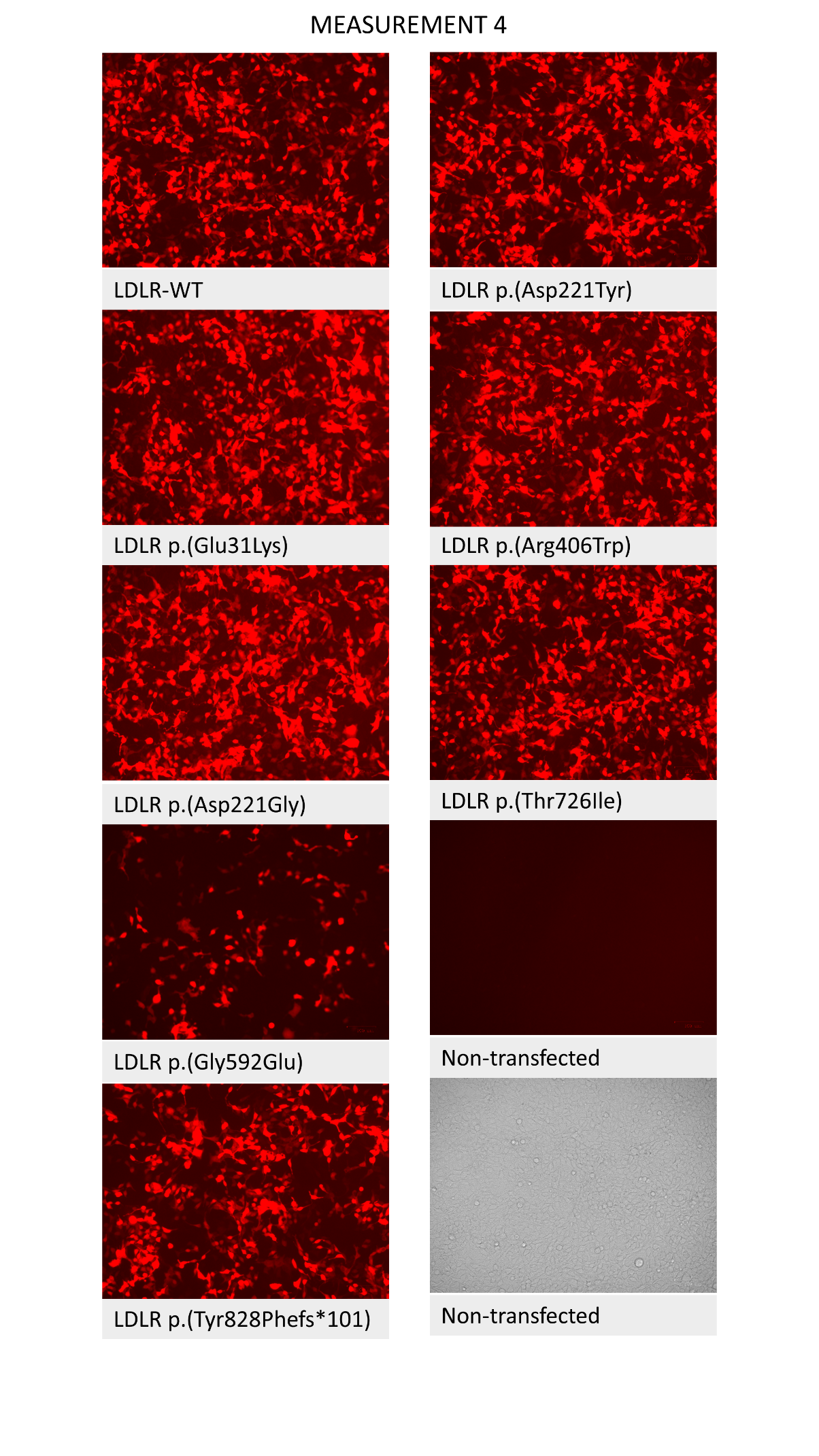
**

**
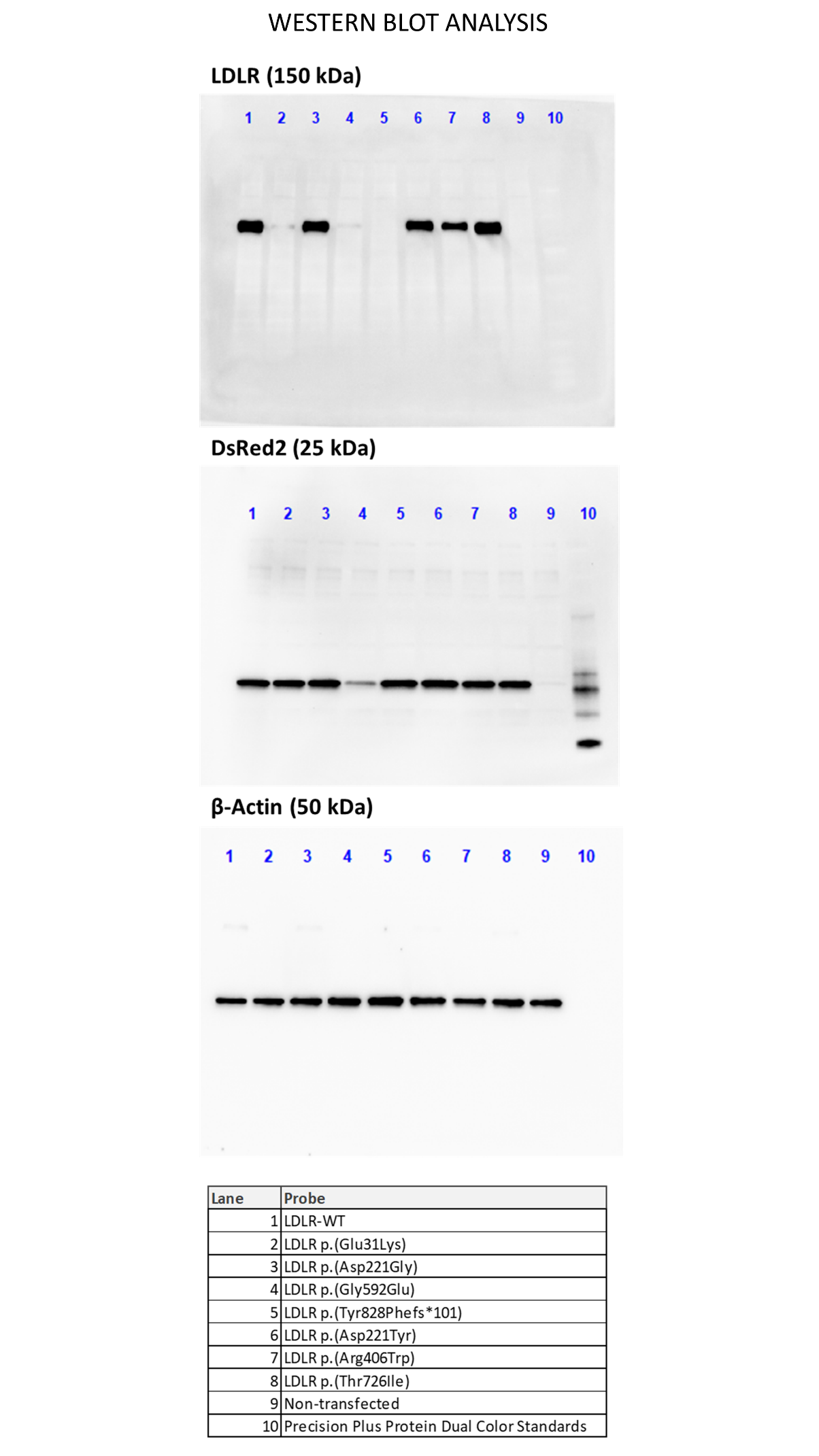
**

**
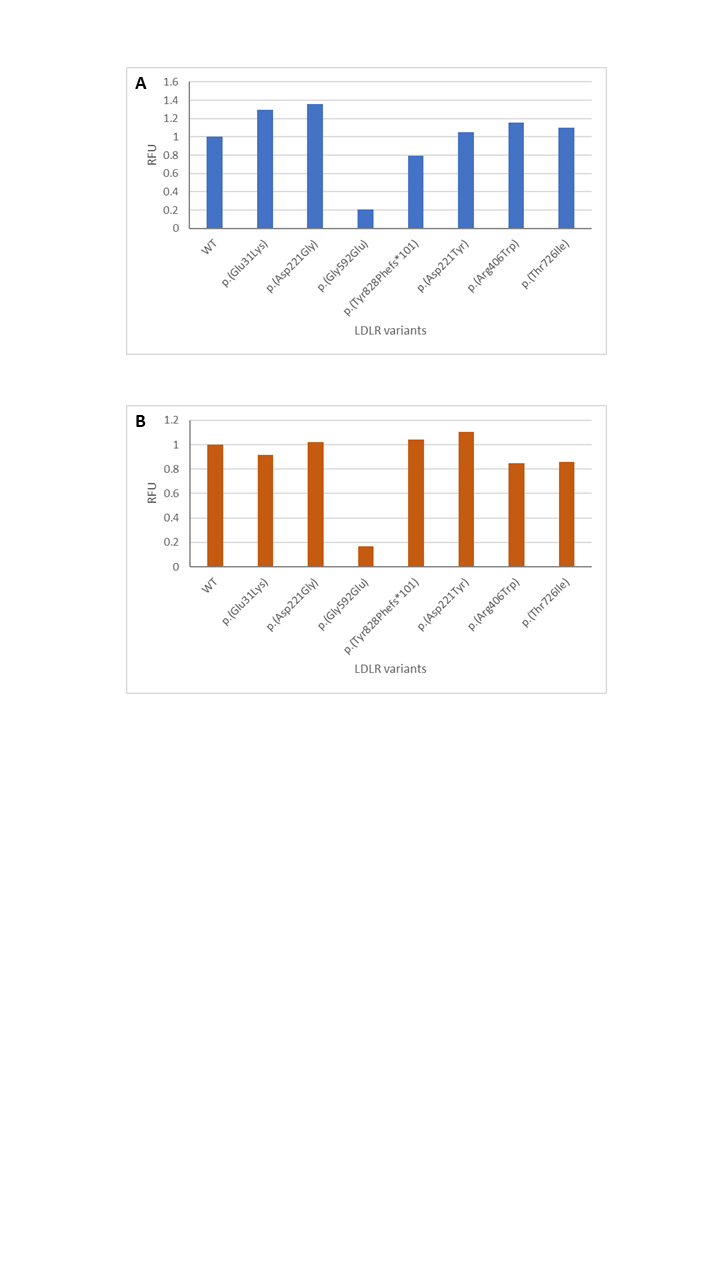
**

**Graph 4 Transfection efficiency measurement 4: (A)** The red fluorescence of DsRed2 was monitored under a fluorescence microscope. Obtained data were calculated using ImageJ software as %Area value^1^ to evaluate the transfection rate. **(B)** The DsRed2 expression in transfected HEK293T-*ldlr*G1 cells was analyzed using a Western blot technique using an anti-DsRed2 primary antibody. The intensity of the bands indicated transfection efficiency and was measured using ImageLab software. LDLR variant expression was normalized to the DsRed2 signal.

^1^ The %Area value is the fraction of the area. For thresholded images, this is the percentage of pixels in the image highlighted in red using the Image-Adjust-Threshold algorithm. Tiago Ferreira, Wayne Rasband; ImageJ User Guide IJ 1.46r

**
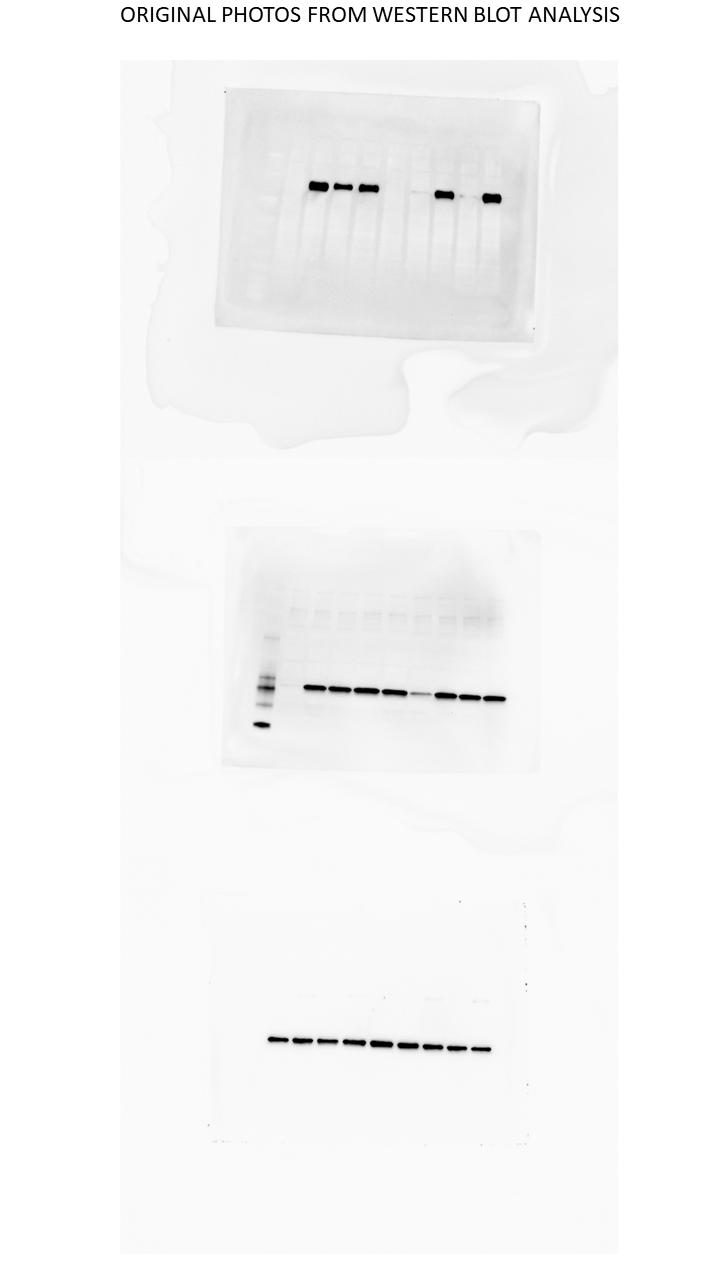
**
